# Endoscopic Management of Mirizzi Syndrome: Experience and Outcomes From a Tertiary Care Center in Mexico

**DOI:** 10.7759/cureus.96694

**Published:** 2025-11-12

**Authors:** Hugo Fernando Narváez González, Rodrigo Soto-Solis, Maricarmen Murillo López, Raúl Sosa Martínez, Laura Olivia Rodríguez Muñoz, Elsa Paulina Alonso López, Astrid Kristel Rojas Hernández, Luis Ariel Waller González

**Affiliations:** 1 Gastrointestinal Endoscopy, Centro Médico Nacional 20 de Noviembre, Instituto de Seguridad y Servicios Sociales de los Trabajadores del Estado (ISSSTE), Ciudad de México, MEX; 2 General Surgery, Hospital de Especialidades de la Cuidad de México Dr. Belisario Domínguez, Ciudad de México, MEX; 3 Gastroenterology, Centro Médico Nacional 20 de Noviembre, Instituto de Seguridad y Servicios Sociales de los Trabajadores del Estado (ISSSTE), Ciudad de México, MEX

**Keywords:** cholangitis, clinical success, endoscopic treatment, mirizzi syndrome, technical success

## Abstract

Introduction: Mirizzi syndrome (MS), an infrequent yet often delayed complication of cholelithiasis, poses diagnostic and therapeutic challenges stemming from its heterogeneous presentation, propensity for fistula formation, and distortion of anatomy within Calot’s triangle. Although surgery remains a reference standard, this study examines therapeutic endoscopy as a safe and effective alternative within a Mexican tertiary center, where disease burden exceeds global estimates.

Methods: We conducted a retrospective, single-center observational study (January 2022-April 2025), identifying 16 patients with MS who underwent 22 endoscopic retrograde cholangiopancreatography (ERCP) procedures (out of 914 total). Data collected included demographics, Mirizzi classification (Csendes), endoscopic approach, technical (successful biliary decompression), and clinical outcomes (avoidance of surgery/complications, resolution of cholangitis). Statistical analyses included Fisher’s exact tests for categorical variables, and Mann-Whitney U tests for continuous variables, with significance set at p < 0.05. Analyses followed established methodological standards.

Results: MS accounted for 2.4% of ERCP volume. Type I predominated (50%), followed by types IV, V, and III. The cohort’s mean age fell in mid-adulthood, without apparent sex predilection. Overall, a technical success rate of 90.9% (20/22 procedures) was achieved, while clinical success was observed in 87.5% (14/16 patients). Non-surgical resolution was accomplished in 75.0% of types IV-V patients. In inferential analyses, procedure duration and fluoroscopy time, when stratified by Mirizzi type or presence of cholangitis (p ≥ 0.05), displayed no statistically significant differences. Advanced techniques, including SpyGlass cholangioscopy, showed a significant association with type IV MS (p = 0.0476). These results demonstrate an improvement as reported in previous success rates.

Conclusion: Therapeutic endoscopy is demonstrably effective and safe for select MS cases, effectively relieving biliary obstruction, and successfully managing cholangitis as either a bridge to eventual surgery or as a definitive standalone solution. Further studies are needed to establish standardized, evidence-based guidelines for endoscopic treatment of this complex syndrome.

## Introduction

Mirizzi syndrome (MS) is a rare and delayed complication of cholelithiasis. It is characterized by extrinsic compression of the common hepatic duct (CHD) or common bile duct (CBD) caused by impacted calculi in Hartmann's pouch, which can lead to the formation of fistulas with adjacent structures. Although the reported worldwide prevalence ranges from 0.7% to 5.7%, and the incidence of cholecystectomies is between 0.3% and 1.4%, studies in Mexico suggest a higher prevalence, approximately 4.7% [1,2].

Acute cholecystitis occurs when gallstones become trapped in the cystic duct or neck of the gallbladder (GB), triggering acute symptoms. Recurrent episodes of obstruction lead to inflammation of the GB and its surrounding structures. When impacted calculi cause partial or complete obstruction of the CHD or CBD, the diagnosis of MS is established [3]. If left untreated, there is a risk of wall erosion, leading to a fistula between the GB and the bile duct (cholecystocholedochal fistula) or a fistula between the GB and the intestine (cholecystoenteric fistula), which may allow stones to migrate through, where they can become impacted, often at the ileocecal valve, and cause mechanical obstruction manifestating gallstone ileus [4].

This pathophysiological process has significant clinical implications, manifesting as right upper quadrant abdominal pain, jaundice, and, in some cases, concomitant cholangitis. However, presentation can often be nonspecific for this syndrome [5].

While surgery remains the first-line treatment, principally to remove the GB and its calculi, this approach can be challenging due to the complexity and the required surgical expertise, particularly because of inflammation within Calot's triangle and distorted anatomy [6]. Surgical options vary from open cholecystectomy to laparoscopic procedures and bail-out techniques, including subtotal (reconstituted or fenestrated) cholecystectomy. These techniques are described as safe and effective; however, potential complications, such as retained stones, bile leaks, and subhepatic or subphrenic collections, must be considered by up to 16% [7].

Supportive surgical procedures, such as intraoperative cholangiography, choledochoplasty, T-tube placement, or bilioenteric reconstruction, are also employed. However, some reports indicate that these interventions are associated with higher rates of conversion to open surgery and greater complication rates, such as biliary duct injury, ranging from 7.7% to 41% [8-10].

Given these challenges, endoscopic procedures offer a valuable alternative with the potential to significantly reduce associated morbidity. Several studies have documented the effectiveness of endoscopic approaches, such as the placement of nasobiliary drains, stent insertion, or advanced lithotripsy techniques, for stone extraction in the biliary tract [11-13].

The objective of this study was to describe the clinical presentation, diagnostic approach, and therapeutic outcomes of patients with MS treated at a tertiary hospital in Mexico, and to analyze outcomes according to the Csendes classification [14].

## Materials and methods

From January 2022 to April 2025, a retrospective, observational, single-center study was approved in accordance with the register RPI.CMN.05-179-2025 and conducted to evaluate the efficacy of endoscopic treatment for MS. During this period, we identified 16 patients who underwent 22 endoscopic retrograde cholangiopancreatography (ERCP) procedures of endoscopic therapy for MS, out of a total of 914 ERCP procedures performed. Data collection was conducted by reviewing medical records, endoscopic reports, and imaging studies from the endoscopy service database. This database is used for patients who are registered after the procedure was completed. We included information on demographic characteristics and the endoscopic report, which provides details of all documented findings, including the material used and both endoscopic and fluoroscopic images documenting the observed pathology. Following the endoscopic procedure, patients were contacted to inquire about the outcome, as some required a subsequent cholecystectomy or other procedures, such as bilioenteric reconstruction in the most complex cases. The inclusion criteria encompassed patients who underwent ERCP at the Centro Medico Nacional 20 de Noviembre, Instituto de Seguridad y Servicios Sociales de los Trabajadores del Estado (ISSSTE), within the study period, were registered in the endoscopy database, and had imaging findings compatible with MS, confirmed via fluoroscopy and ERCP. Exclusion criteria included patients under 18 years of age, undergoing ERCP for indications other than MS, and cases lacking sufficient documentation for analysis. 

Variables analyzed included age, sex, MS classification based on Csendes classification [14], which is the most universally used classification, procedure duration, radiation exposure time, technical success, clinical success, mortality, concomitant choledocholithiasis, cholangitis and endoscopic treatment modality such as cholangioscopy, rendezvous procedure with endoscopic ultrasound (RV-EUS), balloon sweeping, sphincterotomy, mechanical lithotripsy (ML), electrohydraulic lithotripsy (EHL), laser lithotripsy (LL) and biliary stent placement. All procedures utilized a Fujifilm ED-530XT duodenoscope, combined with Boston Scientific accessories, including extraction balloons, baskets, lithotriptors, and single-operator cholangioscopy (SOC) for laser, mechanical, and electrohydraulic lithotripsy. Because there is no standardized approach to treating patients with MS, therapeutic choices were individualized based on case complexity.

Statistical analysis prioritized descriptive reporting given the small sample size (n = 16). Categorical variables are presented as frequencies and percentages [n (%)]. Continuous variables are summarized as median and interquartile range (IQR). Group comparisons were two-sided and exploratory with a nominal significance threshold of p < 0.05; no multiplicity adjustments were applied. Continuous variables were compared using the Mann-Whitney U test (two-sided) due to small sample size and non-normal distributions. Categorical variables were compared using Fisher’s exact test (two-sided), appropriate for sparse tables and small cell counts.

The primary comparative framework contrasted MS type I versus types III-IV for general clinical and procedural outcomes (ERCP duration, fluoroscopy time, cholangitis, technical success, clinical success). In addition, we performed a focused comparison of SOC use between MS type I and MS type IV using Fisher’s exact test (two-sided). P-values are reported as calculated; interpretations emphasize whether findings met the exploratory threshold. Analyses were performed using IBM SPSS Statistics for Windows, Version 28.0 (IBM Corp., Armonk, NY, USA).

## Results

Over more than three years, 914 ERCP procedures were performed by two experienced endoscopists, including 22 (2.4%) for MS in 16 patients. The cohort's mean age was 55.56 ± 13 years, with no significant gender predilection. The average procedure duration was 63.8 ± 33 minutes, with a radiation exposure time of 196 ± 136 seconds. MS type I was the most frequent (8/16, 50%), followed by type IV (5/16, 31.3%), type V (2/16, 12.5%), and type III (1/16, 6.3%). 

Concomitant cholangitis was present in seven patients (43.7%), all of whom received biliary stents to facilitate biliary drainage, resulting in a 100% procedural success rate in stent placement and an improvement in their clinical course. The overall technical success, defined as effective biliary decompression through stent placement or endoscopic clearance of the biliary tract, was 90.9%. Clinical success, characterized by the absence of significant complications and a reduced need for more invasive surgical procedures, was achieved in 87.5% of cases. 

**Figure 1:**
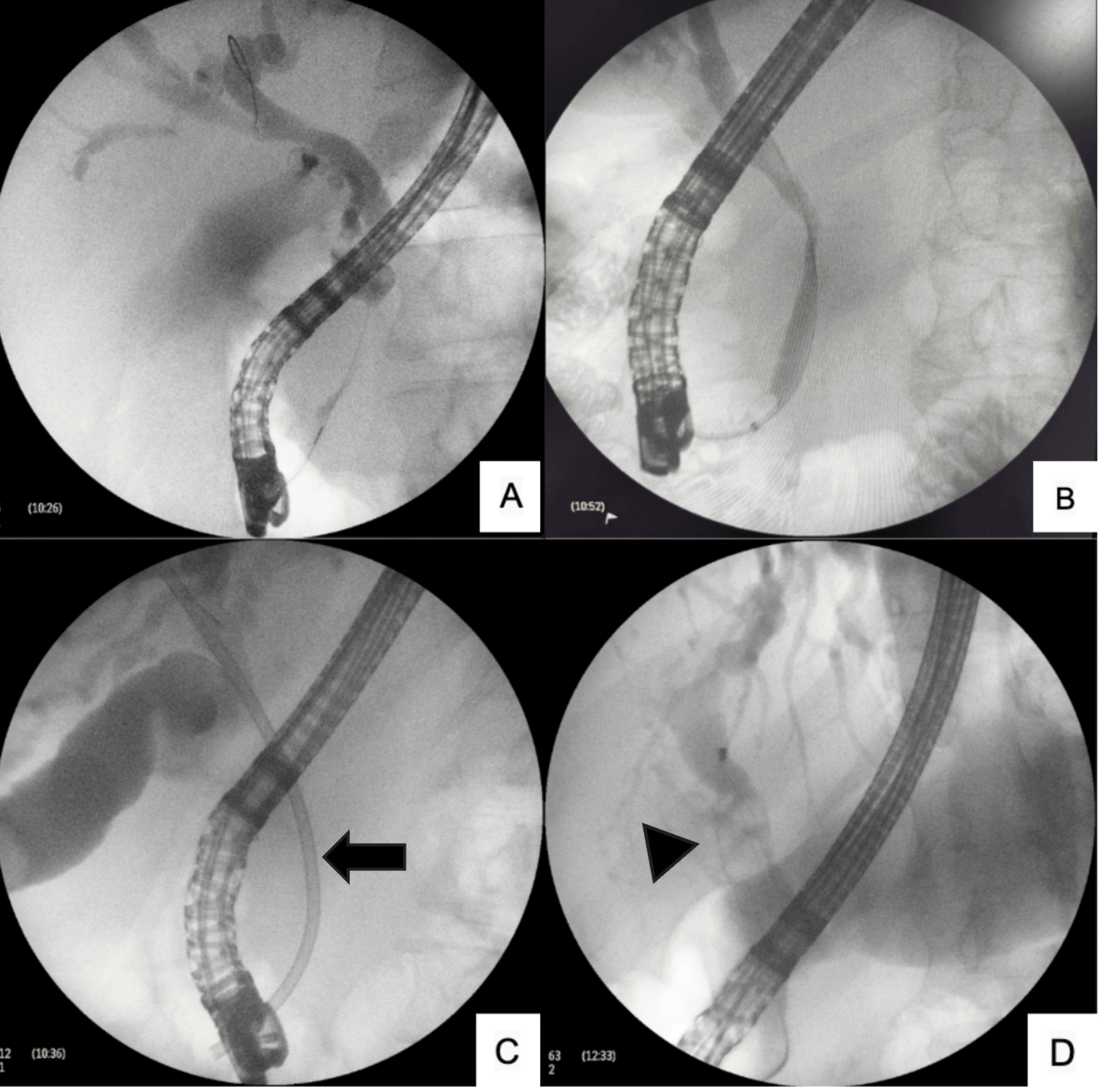
Cholangiographic findings in Mirizzi syndrome. This figure presents cholangiograms from different patients with Mirizzi syndrome. Images A–C correspond to patients with type I MS. Image D depicts a patient with type III MS. In images A and B, the GB is seen extrinsically compressing the bile duct. C) shows a patient with a plastic stent in situ, placed due to concurrent cholangitis. D) illustrates a patient with type III MS and choledocholithiasis in whom multiple attempts at stone extraction with a basket were unsuccessful, ultimately requiring surgical resolution. In image C, the black arrow indicates the plastic stent. In image D, the arrowhead points to the basket and the multiple stones in a patient with type III MS. Abbreviations: MS, Mirizzi Syndrome; GB, Gallbladder.

**Figure 2:**
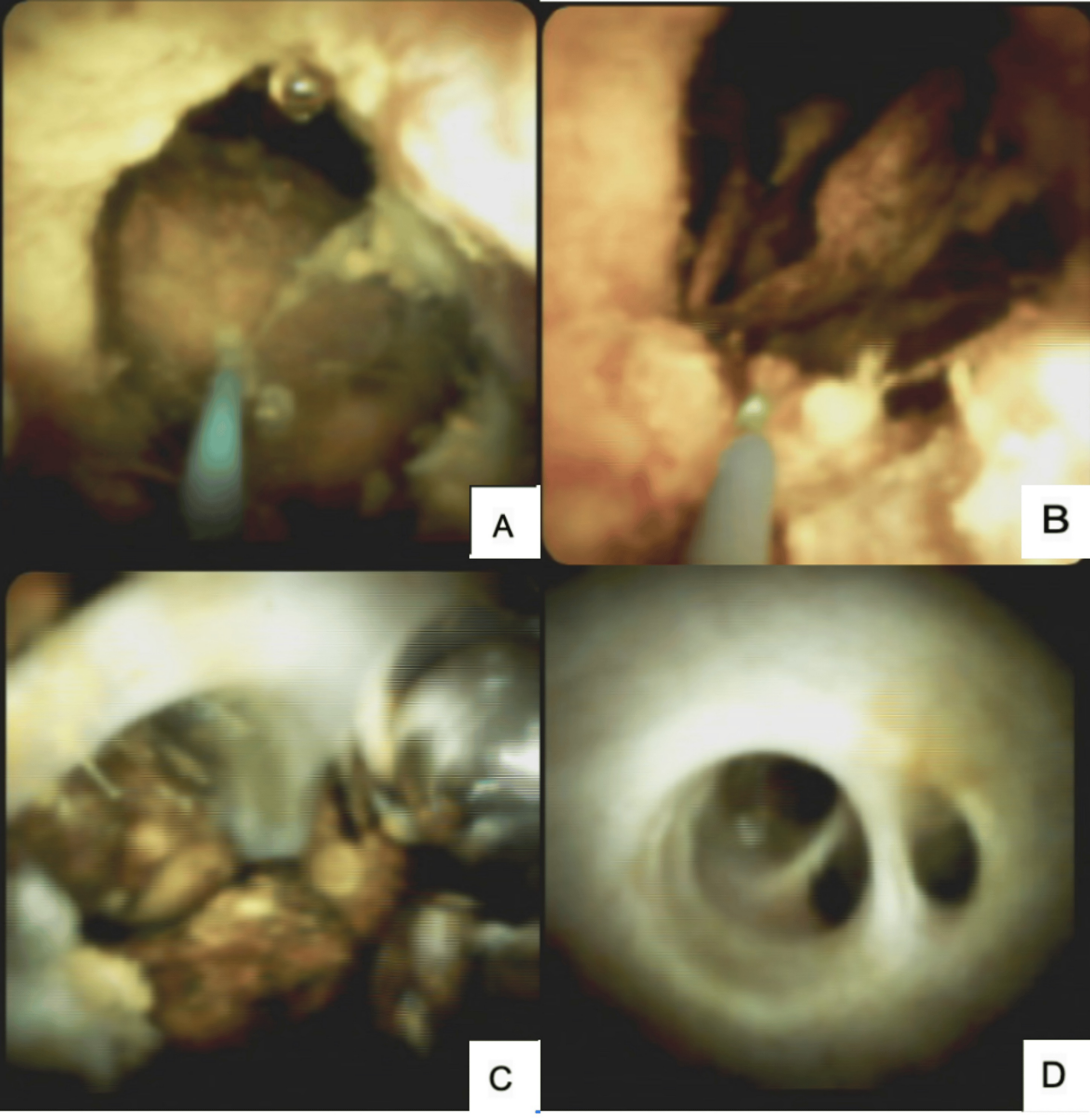
A patient with type IV Mirizzi syndrome underwent cholangioscopy, demonstrating multiple stones within the biliary tract. The stones were fragmented using LL, achieving adequate clearance of the CHD. A) Stones visualized in the CBD. B) LL is employed for stone fragmentation. C) CHD with multiple stones. D) CHD was observed after EHL and LL were performed following stone fragmentation. Abbreviations: LL, Laser Lithotripsy; CBD, Common Bile Duct; CHD, Common Hepatic Duct; EHL, Electrohydraulic Lithotripsy.

The intervention variables and MS types are summarized in Table 1. Most patients required biliary stent placement (n=15, 68.1%) and ML (n=9, 40.9%). The stents employed were predominantly plastic in 59% and metallic in 9%. Figure 1 shows cholangiograms from selected patients with predominantly type I MS, demonstrating extrinsic compression of the biliary duct by the GB, as well as the use of devices such as a stone extraction basket in a patient with type III. Four patients with MS type IV who were candidates for a bilioenteric reconstruction, given the size of the fistula, circumferential involvement of the CBD, and difficulty achieving by ML, underwent SOC using SpyGlass, with successful use of EHL and LL (see Figure 2). SOC was used exclusively in patients with type IV MS because it was not available for use in patients with types III and V at the time of their treatment.

**Table 1:** Patient characteristics, interventions, and outcomes by Mirizzi syndrome type following endoscopic management Characteristics, treatments, and outcomes for patients with Mirizzi syndrome managed endoscopically. Data include demographics, Mirizzi syndrome type, endoscopic interventions performed, and success rates. For categorical variables, 'N' represents the number of patients/procedures, and '%' is the percentage of the total group, except where otherwise indicated. Percentages for MS subtypes indicate the proportion of each subtype within the total patient cohort (N = 16). Success rates for the overall patient group are marked with a single asterisk (*). Success rates within each Mirizzi subtype are marked with double asterisks (**). Abbreviations: MS, Mirizzi Syndrome; ERCP, Endoscopic Retrograde Cholangiopancreatography; SOC, Single-Operator Cholangioscopy; EUS, Endoscopic Ultrasound; EHL, Electrohydraulic Lithotripsy; LL, Laser Lithotripsy; BER-HJ, Bilioenteric Reconstruction with Hepaticojejunostomy; N/A, Not Applicable.

Variables	Total N (%)	MS Type I N (%)	MS Type III N (%)	MS Type IV N (%)	MS Type V N (%)
Patients	16 (100.0)	8 (50.0)	1 (6.2)	5 (31.3)	2 (12.5)
Number of ERCPs	22 (100.0)	11 (50.0)	1 (4.5)	8 (36.4)	2 (9.1)
Gender - Male	8 (50.0)	4 (25.0)	0 (0.0)	3 (18.8)	1 (6.2)
Gender - Female	8 (50.0)	4 (25.0)	1 (6.2)	2 (12.5)	1 (6.2)
Age (years)	55.6 ± 13.0	57.1 ± 13.4	60.0 ± N/A	52.0 ± 13.0	62.5 ± 2.1
Cholangitis	7 (43.7)	7 (43.7)	0 (0.0)	0 (0.0)	0 (0.0)
Choledocholithiasis	10 (62.5)	3 (18.8)	1 (6.2)	5 (31.2)	1 (6.2)
Stent Placement	15 (68.2)	9 (40.9)	0 (0.0)	5 (22.7)	1 (4.6)
Plastic stent	13 (59.1)	9 (40.9)	0 (0.0)	3 (13.6)	1 (4.6)
Metal stent	2 (9.1)	0 (0.0)	0 (0.0)	2 (9.1)	0 (0.0)
SOC	4 (18.2)	0 (0.0)	0 (0.0)	4 (18.2)	0 (0.0)
EUS	1 (4.5)	0 (0.0)	0 (0.0)	1 (4.5)	0 (0.0)
Sphincteroplasty	1 (4.5)	0 (0.0)	0 (0.0)	0 (0.0)	1 (4.5)
Mechanical Lithotripsy	9 (40.9)	3 (13.6)	1 (4.5)	3 (13.6)	2 (9.1)
EHL	1 (4.5)	0 (0.0)	0 (0.0)	1 (4.5)	0 (0.0)
LL	1 (4.5)	0 (0.0)	0 (0.0)	1 (4.5)	0 (0.0)
BER - HJ	2 (25.0)	0 (0.0)	1 (12.5)	1 (12.5)	0 (0.0)
Non-surgical Resolution	6 (75.0)*	N/A	0 (0.0)	4 (80.0)**	2 (100.0)**
Technical Success	20 (90.9)*	8 (72.7)**	0 (0.0)	4 (50.0)**	2 (100.0)**
Clinical Success	14 (87.5)*	8 (100.0)**	0 (0.0)	4 (80.0)**	2 (100.0)**
Mortality	1 (6.2)	0 (0.0)	0 (0.0)	0 (0.0)	1 (6.2)

**Figure 3:**
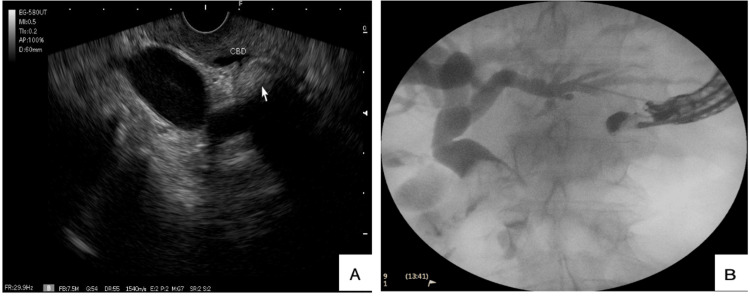
Patient with type IV MS with total cholecystobiliary fistula and multiple stones identified by fluoroscopy, causing dilation of intrahepatic and extrahepatic biliary ducts. The patient required BER-HJ. A) Visualized stones on EUS with dilation of the biliary tract. The white arrow points to stones within the bile duct. B) Antegrade cholangiography via EUS showing multiple stones in the CBD. Abbreviations: MS, Mirizzi Syndrome; CBD, Common Biliary Duct; EUS, Endoscopic Ultrasound; BER-HJ, Bilioenteric Reconstruction with Hepaticojejunostomy.

In a patient with MS type IV, attempts were made to perform RV-EUS; however, cannulation via antegrade and retrograde approaches was unsuccessful (see Figure 3). Patients in whom endoscopic resolution was unfeasible required bilioenteric reconstruction. One patient with MS type V died due to causes unrelated to the procedure.

In exploratory two-sided comparisons between MS type I and types III-IV, there was no statistically significant difference in ERCP duration (Mann-Whitney U; p = 0.399) or fluoroscopy time (Mann-Whitney U; p = 0.329). Similarly, the proportions of patients with cholangitis did not differ significantly between groups (Fisher’s exact test; p = 0.301), and there were no significant differences in technical success (Fisher’s exact test; p = 0.209) or clinical success (Fisher’s exact test; p = 0.277); overall success rates were high. In a focused analysis comparing SOC use between MS type I and MS type IV, we observed a statistically significant difference (Fisher’s exact test; p = 0.0476), indicating greater use in type IV than in type I and suggesting that this advanced technique is handy in more complex cases with cholecystocholedochal fistulas. The exploratory comparisons between MS type I and types III-IV are summarized in Table 2. Given the small sample size and the exploratory intent, findings should be interpreted cautiously and considered hypothesis-generating.

**Table 2:** Exploratory comparisons between Mirizzi syndrome subtypes (two-sided tests) Exploratory analysis with two-sided tests at p < 0.05. P-values are unadjusted and should be interpreted cautiously, given the small sample size. Tests used: Mann–Whitney U for continuous variables; Fisher’s exact test for categorical variables. The cholangioscopy comparison reflects the provided counts, indicating use in type IV and none in type I. Abbreviations: MS, Mirizzi Syndrome; ERCP, Endoscopic Retrograde Cholangiopancreatography; SOC, Single-Operator Cholangioscopy; min, minutes; s, seconds.

Comparison	Variable	Groups	Statistical Test	P-value
MS Type (I vs III–IV)	ERCP duration, min	I vs III–IV	Mann–Whitney U (two-sided)	0.399
MS Type (I vs III–IV)	Fluoroscopy, s	I vs III–IV	Mann–Whitney U (two-sided)	0.329
MS Type (I vs III–IV)	Cholangitis (yes/no)	I vs III–IV	Fisher’s exact test (two-sided)	0.301
MS Type (I vs III–IV)	Technical success (yes/no)	I vs III–IV	Fisher’s exact test (two-sided)	0.209
MS Type (I vs III–IV)	Clinical success (yes/no)	I vs III–IV	Fisher’s exact test (two-sided)	0.277
MS Type (I vs IV)	SOC (yes/no)	I vs IV	Fisher’s exact test (two-sided)	0.0476

Beyond technique, these findings highlight a contemporary care model uniting technical precision and practice, offering a minimally invasive pathway that both respects altered anatomy and safeguards more fragile patients, while partnering seamlessly with surgical interventions when such become strictly required.

## Discussion

The first description of MS was documented in 1905 by Hans Kehr. However, it was Pablo Luis Mirizzi who formally named it "Mirizzi syndrome" in 1948 [8,14]. The pathophysiology of MS has been summarized by Csendes et al. and McSherry et al., detailing the progression from impacted gallstones to erosion of the GB wall and the CBD, ultimately leading to the formation of a cholecystobiliary fistula and the development of additional fistulas to adjacent viscera, such as the duodenum, stomach, and colon [1,14-16].

Jun Wu and colleagues highlight various factors contributing to MS development; typically, the GB exhibits increased size, thickened walls, and elevated pressure, with significant edematous and inflammatory tissue in Calot's triangle secondary to stone impaction. This promotes the formation of a cholecysto-biliary and/or cholecysto-enteric fistula [17]. The stone migrates toward the CBD or intestine, leading to decompression of the GB, which contracts and reduces in size. Inflammatory edema of the GB and Calot’s triangle results in fibrosis, with the fistula often occluded by an impacted stone at its opening [7]. Several classifications have been proposed for evaluating MS, but the one established by Csendes et al. in 1989 remains the most widely accepted and validated [5,14]. Type I refers to external compression of the bile duct caused by an impacted stone at the GB neck or cystic duct. Types II through IV involve a cholecystobiliary fistula affecting one-third, two-thirds, or the entire circumference of the bile duct wall, respectively. Type V is associated with a cholecystoenteric fistula, with or without gallstone ileus [5,7].

The management of MS has evolved considerably over time. Historically, definitive treatment relied on open surgery. Later, laparoscopic approaches were adopted, but these have been associated with notable conversion rates to open procedures and biliary injuries, reaching up to 30% and 14%, respectively [18-20]. Since the development of cholangioscopes in the 1970s, initially requiring two trained endoscopists due to the "mother-baby" endoscope technique, the management of bile duct stones has evolved toward a less invasive approach [21]. In a 2022 case report, the first successful resolution of type IV MS was achieved using SOC and EHL, with no complications, and a one-year follow-up showed no recurrence of stones [22].

Another study by Salgado-Garza et al. described their experience with three MS patients managed endoscopically using SOC combined with lithotripsy. In two cases, more than one session of lithotripsy was necessary for adequate stone fragmentation; none of the patients experienced complications. The authors concluded that this approach is a safe and effective alternative, with complication rates lower than traditional methods and a success rate ranging from 90% to 100% [23].

Furthermore, Tsuyuguchi et al. conducted a long-term follow-up of 122 patients with difficult stones, including 47 with type II MS, all of whom underwent cholangioscopy and lithotripsy. The study reported a 95.9% successful removal rate, including all cases of type II MS [24].

In a retrospective study conducted at Ankara Education and Research Hospital, 515 patients underwent ERCP, of whom 12 presented with MS and all required biliary stent placement. Seven of these patients had type III MS. The majority were female, with a mean age of 45 ± 24 years. 66.6% of MS cases required cholecystectomy and T-tube placement, while the remaining patients underwent just laparoscopic cholecystectomy, without postoperative complications. These findings suggest that stent placement may help reduce the risk of biliary injury [25].

In 2022, Pawa Rishi et al. published a retrospective cohort study from the United States comprising 5,123 ERCP procedures. Among these, 21 patients were diagnosed with cystic duct stones, and five with type I MS. Cholangioscopy-EHL was utilized in three of these patients (representing 20% of all cases undergoing cholangioscopy). At the same time, the other two were treated with balloon extraction. The success rate in these cases was 100% [12].

In our study, 16 patients were included, with the majority presenting with type I MS (50%), followed by five patients (22.7%) with type IV, and the remaining cases consisting of two patients with type V and one with type III MS. This distribution contrasts with another Mexican study, where type I was the most prevalent, accounting for 90% of the cohort [26].

Biliary stents were placed in 15 procedures (68.1%) to achieve adequate biliary drainage and as part of the management of cholangitis, which was observed exclusively in patients with type I MS. Similar to the findings of Karaahmet et al., in our population, stents were employed in all patients with type I MS, who subsequently underwent laparoscopic cholecystectomy in a staged approach without complications [8,25]. Biliary stenting in patients with MS may reduce the risk of complications, which have been demonstrated to be higher during cholecystectomy. Nonetheless, further research is necessary to determine whether the observed benefit primarily stems from resolving biliary obstruction or involves other factors related to MS.

The 2019 guidelines from the American Society for Gastrointestinal Endoscopy state that ERCP may serve as an alternative to surgical treatment in MS, contingent upon the clinician’s expertise [27]. Similarly, they note that ERCP alone has a modest success rate of approximately 40% when using conventional techniques, with successful outcomes increasing to 75%-91% when combined with cholangioscopy [24,28,29].

Our technical and clinical success rates exceeded 87% in achieving MS resolution, comparable to other cohorts in which techniques such as mechanical lithotripsy, functional bypass with stent placement, and advanced methods are fundamental for adequate stone clearance [12,23,24]. However, our study included a limited number of patients; therefore, caution is warranted when interpreting the generalizability of the reported technical and clinical success.

Recently, a review comparing surgical versus SOC in the management of types II-IV MS in 290 patients demonstrated that the surgical approach had a higher technical success rate (96.5% vs. 89.8%, p = 0.035). However, SOC exhibited a significantly lower overall complication rate (10.2% vs. 41.2%, p<0.001), reduced need for hepaticojejunostomy (8.2% vs. 25.4%, p=0.006), and a lower risk of conversion to open surgery in patients who previously underwent SOC (6.0% vs. 22.8%, p=0.009) [30]. These findings provide additional evidence supporting the safety benefits of endoscopic approaches in MS management, consistent with our results showing a decreased need for hepaticojejunostomy in patients with MS types that render them candidates for this procedure. However, it is essential to acknowledge that long‑term outcomes following treatment of MS types III-V remain to be characterized to determine whether these patients ultimately require surgical intervention or can be managed solely with prolonged surveillance. This represents an important avenue for future investigation.

Strengths and limitations of the study

Our study presents certain limitations and areas for improvement. Firstly, its retrospective design introduces potential selection bias. Secondly, the sample size is small, so it would be advisable to continue the study to extend the observation period and capture a larger patient cohort. Additionally, we did not perform long-term follow-up, which would be instrumental in determining the recurrence risk or disease-free interval associated with endoscopic treatment compared to surgical intervention. Unfortunately, there is still no standardized endoscopic approach for treating MS. Consequently, further research is necessary to gather more information on experience and identify the most effective techniques to minimize complications, reduce costs, and decrease the need for surgical procedures. 

Among the study's strengths, we note that it is an investigation conducted in Mexico evaluating patients with MS treated via an endoscopic approach, employing both conventional and advanced techniques across the spectrum from type I to type V MS, except type II. Finally, we recognize that endoscopic management offers a significant alternative for patients, making it an excellent therapeutic option in carefully selected cases, particularly because it can reduce the risk of bile duct injury during cholecystectomy or avoid a bilioenteric reconstruction in those with more advanced MS. Furthermore, this study adds to the growing body of evidence supporting an alternative treatment approach for patients who are suitable candidates for endoscopic management. As such, it strengthens the foundation for future research in this area.

Finally, it is essential to note that we use the term "challenges" because, despite advances in technology, there are cases where endoscopy cannot provide adequate treatment. Surgery will remain necessary in the management of this condition. However, some cases can be successfully treated endoscopically. Ultimately, a multidisciplinary approach is the optimal strategy for achieving the best possible outcomes for our patients.

## Conclusions

This study demonstrates endoscopic intervention as a safe and effective strategy for MS, achieving high technical (90.9%) and clinical (87.5%) success rates through both conventional and advanced techniques. Endoscopic management offers a less invasive, viable alternative, potentially reducing the need for more invasive procedures and improving patient quality of life, particularly with advanced techniques enabling treatment of complex cases while avoiding bilioenteric reconstruction in 75% of patients. These findings support a contemporary multidisciplinary model - a minimally invasive approach respecting anatomy and patient safety, partnering with surgery strategically. However, definitive clinical guidelines and optimized outcomes require multicenter prospective studies with long-term follow-up in this complex pathology.
